# The association between exposure to food marketing and dietary intake among youth in six countries

**DOI:** 10.1186/s12966-025-01828-2

**Published:** 2025-10-21

**Authors:** Laura Vergeer, Grace Gillis, Vicki L. Rynard, Lana Vanderlee, Christine M. White, Claudia Nieto, David Hammond, Monique Potvin Kent

**Affiliations:** 1 School of Epidemiology and Public Health, Faculty of Medicine, University of Ottawa, Ottawa, ON Canada; 2 School of Public Health Sciences, University of Waterloo, Waterloo, ON Canada; 3 École de Nutrition, Centre de Nutrition, santé et société (NUTRISS), Université Laval, Québec City, Québec Canada; 4 Department of Public Health, Faculty of Medicine, National Autonomous University of Mexico, Mexico City, Mexico

**Keywords:** Food marketing, Less healthy food, Dietary intake, Youth

## Abstract

**Background:**

While food marketing to youth is associated with harmful behavioural and dietary outcomes, few studies have assessed differences in this relationship between countries. This study examined the association between exposure to food marketing and dietary intakes among youth in six countries.

**Methods:**

A cross-sectional analysis of International Food Policy Study 2023 Youth Survey data examined the relationship between self-reported exposure to marketing for less healthy (fast food, sugary drinks, sugary cereals, snacks, desserts/treats) and healthy (fruits, vegetables) food categories across various media/settings in the past 30 days and consumption of these foods yesterday among youth 10-17 years-old in Canada, Australia, Chile, Mexico, the United Kingdom and the United States (*n*=9057). Associations of food consumption with exposure to marketing of food categories and marketing techniques (e.g., characters, famous people) in food advertisements, and differences in associations between countries, were examined using binary and ordinal logistic regression.

**Results:**

In all countries, youth reporting more frequent exposure to marketing of all less healthy food categories had higher odds of having consumed those foods yesterday (*p* < 0.05 for all), except snacks in Mexico. Compared with no exposure to marketing techniques, exposure to ≥ 1 marketing technique(s) in less healthy food advertisements was associated with higher odds of having consumed sugary drinks (AOR: 1.44; 95% CI: 1.21, 1.72), fast food (AOR: 1.69; 95% CI: 1.40, 2.03), sugary cereals (AOR: 1.26; 95% CI: 1.05, 1.51) and desserts/treats yesterday (AOR: 1.42; 95% CI: 1.18, 1.71) among youth in all countries. Consumption of snacks was associated with exposure to ≥ 1 marketing technique(s) in less healthy food advertisements in Australia (AOR: 1.61; 95% CI: 1.09, 2.34), Chile (AOR: 1.63; 95% CI: 1.12, 2.36) and Mexico (AOR: 2.13; 95% CI: 1.39, 3.26). Positive associations between frequency of exposure to marketing of fruits and vegetables and the number of times these foods were consumed yesterday were observed in all countries (*p* < 0.05), except vegetable consumption in the UK.

**Conclusions:**

These results support the association between exposure to food marketing and consumption of marketed foods. Findings were similar between countries, reinforcing the need for global implementation of restrictions on food marketing to youth.

**Supplementary Information:**

The online version contains supplementary material available at 10.1186/s12966-025-01828-2.

## Background

Noncommunicable diseases, including obesity, heart disease, cancer, and diabetes, are the leading cause of global mortality and morbidity, accounting for 74% of all deaths worldwide [1]. Between 1975 and 2016, the proportion of children and adolescents (hereafter referred to as “youth”) classified as having overweight or obesity increased from 4% to over 18%, affecting more than 550 million individuals aged 5 to 19 years worldwide [2, 3]. Noncommunicable diseases are driven by a complex interplay of individual behaviours, social influences, and environmental conditions, with poor diet quality identified as a key contributor [3, 4]. In Canada, youth aged 2 to 18 years consume over 50% of their daily energy from ultra-processed foods, such as fast food, packaged snacks, desserts and sugary drinks [5]. These products are typically high in nutrients of concern including sodium, sugars, and/or saturated fats, and low in essential nutrients such as fibre, protein, and micronutrients [6]. Likewise, between 2004 and 2015, Canadian youth experienced an overall decline in fruit and vegetable intake and consistently consumed fewer than the recommended number of servings [7]. Given the critical role of diet in shaping long-term health outcomes, understanding the factors that influence food choices, such as exposure to food marketing, is essential for developing effective strategies to reduce the burden of noncommunicable diseases.

Marketing of less healthy foods and beverages has been identified as a contributor to poor diet quality [8]. Systematic reviews have shown that exposure to food and beverage marketing strongly influences youth food preferences, choices, and purchases (or purchase requests) [8]. This is particularly concerning given that most food products marketed to children are nutrient-poor and energy-dense [9]. Children are especially vulnerable to persuasive marketing techniques (e.g., spokes characters, celebrities, promotional offers), given they are still undergoing cognitive development, have a limited ability to understand the persuasive intent of advertising, and are often unable to distinguish advertisements from non-commercial content [10–12]. Youth encounter food advertisements through a variety of traditional and digital media and settings, such as television, social media, food packaging, and retail environments, among others [13]. For example, a research has estimated Canadian youth aged 6–17 years are exposed to 6023 food ads/child/year via digital media, while Mexican youth’s digital food marketing exposure was estimated as 2461 marketing instances per year [14, 15]. In addition, studies from Canada, the US and the UK have shown that youth’s exposure to food advertisements on television remains high [16–18]. While most food marketing research has focused on television and digital media, other media and settings, such as food packaging, outdoor marketing, restaurant and retail point-of-sale, recreation centres and sporting events, are also important contributors to youth’s marketing exposure [19–23].

A growing body of evidence shows that youth exposed to marketing for less healthy food and beverages are more likely to consume these products [10, 24–31]. A systematic review of 29 international studies involving children aged 2 to 18 years found that exposure to less healthy food marketing increased both immediate dietary intake and the likelihood of selecting advertised products during or shortly after exposure to advertisements [10]. This association has been demonstrated in various media and settings [10, 13, 29, 31]. For example, studies have shown more frequent self-reported exposure to marketing of fast food and sugary drinks are associated with reporting more frequent consumption of these foods among youth from Canada, Australia, Chile, Mexico, the UK and US [30, 32]. Such findings underscore the need to further examine how exposure to food marketing influences dietary patterns, including the consumption of both less healthy foods and healthy foods like fruits and vegetables. The relationship between exposure to fruit and vegetable marketing and consumption of these foods among youth has not been well established, but there is some evidence of a positive relationship [33–35].

In response to the impacts of food and beverage marketing on children, the World Health Organization has called for member states to implement mandatory restrictions [36]. In most countries with regulations, marketing policies are a combination of statutory and/or self-regulatory measures, and typically only protect children under the age of 13 [37]. Canada (excluding the province of Quebec), Australia, and the United States rely on industry self-regulation [38–40]. Extensive research has shown that self-regulatory approaches are largely ineffective at meaningfully reducing children’s exposure to food advertisements [17, 39, 41–44]. In Quebec, the Consumer Protection Act is mandatory provincial legislation that prohibits all commercial advertising directed at children under the age of 13. Mandatory food marketing restrictions also exist in Mexico and Chile across multiple media and settings. Additionally, in the UK, the promotion of products high in saturated fat, salt and sugar during children’s television programming (i.e., for those aged 4–15 years) has been prohibited since 2007 and will be expanded to include all TV programming (airing before 9 pm) and paid-for online advertising as of January 2026 [45]. Mexico’s policy consists of restrictions on food advertising on television and in cinemas directed at children under 13 years-old (where ≥ 35% of the audience consists of children at certain times of day), and includes food category-specific thresholds for energy, sodium, saturated fat and total sugars that advertised products must not surpass [46]. Child-appealing packaging is also prohibited on foods with front-of-package warning labels on foods with excess calories, sodium, saturated fat, trans fat or sugars, and/or foods with caffeine or artificial sweeteners content. Chile also has food marketing legislation that restricts marketing of foods with warning labels indicating high in calories, saturated fat, sodium or sugars directed at children under 14 years-old on TV, digital media, food packaging and in schools [46]. In the United Kingdom, legislation has been passed that will restrict television food advertising between 5:30 am and 9 pm and ban all paid ads online as of January 2026 [45].

The impact of these regulations on youth’s marketing exposure and food consumption has not been fully studied. Understanding the frequency and types of food marketing to which youth report being exposed to and the impacts of this marketing on dietary behaviours are critical to informing more effective policy interventions. As such, this study aimed to examine the association between self-reported exposure to food marketing across various media/settings and consumption of less healthy foods and fruits and vegetables among youth from six countries with differing regulatory landscapes concerning food marketing.

## Methods

Data were from the 2023 International Food Policy Study (IFPS) Youth Survey, an annual repeat cross-sectional survey conducted in Australia, Canada, Chile, Mexico, the United Kingdom and the United States [47]. Data were collected via self-completed web-based surveys conducted in November-December 2023 with youth aged 10 to 17 years. Respondents were recruited through parents/guardians enrolled in the Nielsen Consumer Insights Global Panel and their partners’ panels. Email and panelist dashboard application invitations with unique survey links were sent to adult panelists within each country. Those who confirmed they had a child aged 10 to 17 years living in their household were asked for permission for their child to complete the survey (only one child per household was invited). Children aged 10 to 17 years were eligible to participate, with quotas for age and sex groups in the United Kingdom and United States (given the relatively large panels in these two countries). After eligibility screening, all potential respondents were provided with information about the study and asked to provide assent. Surveys were conducted in English in Australia and the United Kingdom; Spanish in Chile and Mexico; English or French in Canada; and English or Spanish in the United States. Members of the research team who were native in each language reviewed the French and Spanish translations independently. The median survey time was 23 min. The child’s parent/guardian received remuneration in accordance with their panel’s usual incentive structure (e.g., points-based or monetary rewards, chances to win prizes). The study was reviewed by and received ethics clearance through a University of Waterloo Research Ethics Board (REB# 41477) as well as the University of Ottawa Research Ethics Board (H-06-20-5908). A full description of the study methods can be found in the International Food Policy Study: Technical Report – 2023 Youth Survey [48].

### Measures

All survey questions (except age and sex) included the response options “don’t know” and “refuse to answer”.

#### Consumption of less healthy foods and fruits and vegetables

Participants reported whether they had consumed each of the following foods yesterday (by indicating “yes” or “no”): sugary drinks; fast food from a restaurant; sugary cereals; snacks like crackers, chips or granola bars; and desserts or treats like cookies, ice cream or candy. Participants were also asked how many times they had eaten fruits yesterday, using response options ranging from 0 to 10 times. Similarly, participants reported how many times they had eaten vegetables during the previous day, ranging from 0 to 10 times. Due to low numbers of observations at the higher end of the scale, the fruit and vegetable consumption responses were recoded as follows: 0 times; 1 time; 2 times; and 3 or more times.

#### Food marketing exposure

Participants were asked to report how often they had seen or heard advertisements for the following types of foods or drinks in the previous 30 days: sugary drinks; fast food from a restaurant; sugary cereals; fruit or vegetables; snacks like crackers, chips or granola bars; or desserts or treats like cookies, ice cream or candy. Response options included “never”, “less than once a week”, “once a week”, “a few times a week”, “every day” and “more than once a day”. For the regression analyses examining fruit, vegetable or snack consumption as outcomes and stratified by country, the “every day” and “more than once a day” frequency of advertisement exposure categories were combined (“every day or more than once a day”) due to small numbers of observations. Additionally, exposure to specific marketing techniques was assessed by asking participants whether they had seen less healthy food or drinks advertised in the last 30 days with any of the following (by indicating “yes” or “no”): sports teams or athletes; cartoons or characters from movies or TV (e.g., superheroes, Disney); cartoons or characters made by food companies (e.g., Tony the Tiger, Ronald McDonald); famous people. To examine associations with these marketing techniques, a new binary variable was created to indicate whether each participant reported exposure to less healthy food advertisements featuring one or more of the four marketing techniques in the 30 days preceding the survey.

While neither the frequency of food marketing exposure nor the marketing techniques questions specified media or settings of exposure, the preceding survey question asked participants where they had seen or heard advertisements for unhealthy foods or drinks in the last 30 days, with the following media/settings included as response options: TV shows, series or movies; website or social media; video or computer games; stores (such as posters, special displays); radio; magazine or newspaper; billboard; buses, bus stops and other public transit; movie theatres; school; recreation or community centre; and sports event, concert or community event. It is therefore presumable that participants had these media/settings in mind when answering the questions about frequency of food marketing exposure and exposure to marketing techniques.

#### Sociodemographic characteristics

Participants reported their sex at birth (male or female). They also indicated their age, with response options ranging from 10 years to 17 years in 1-year increments, which was collapsed into two categories (10–12 years and 13–17 years) given that existing and proposed government and industry policies concerning marketing to children typically apply to those under 13 years of age [36]. Adapted census measures tailored to each country were used to assess ethnicity, with participants categorized as ‘majority’ if they identified as ‘White’ (Canada, UK, and USA), predominantly English-speaking (Australia), or non-Indigenous (Mexico and Chile) (criteria terminology differed by country depending on what was most appropriate). Perceived income adequacy was assessed by asking parents how easy it is for them to “make ends meet” based on their monthly income by selecting one of the following response options: “very difficult”; “difficult”; “neither easy nor difficult”; “easy”; or “very easy” [49].

### Data analysis

A total of 12,065 children completed the survey. Respondents were excluded for the following reasons: ineligible region; invalid response to a data quality question; below a minimum survey completion time based on median survey time (see Technical Report for details) [48]; and/or multiple invalid responses to open-ended measures (*n* = 544). The analytic sample included 11,521 respondents (Australia: *n* = 1279; Canada: *n* = 3846; Chile: *n* = 1567; Mexico: *n* = 1586; United Kingdom: *n* = 1603; United States: *n* = 1640). A sub-sample of 9057 participants were included in the current analysis after excluding respondents who responded “don’t know” or “refuse to answer” to the questions about food consumption, food marketing exposure or sociodemographic characteristics of interest (*n* = 2464). Data were weighted with post-stratification sample weights constructed using a ranking algorithm with population estimates from the census in each country based on age group, sex, region, and ethnicity (except in Canada). Estimates reported are weighted unless otherwise specified.

Separate binary logistic regression models were used to examine the odds of having consumed sugary drinks, fast food, sugary cereals, snacks, or desserts/treats in the day preceding the survey, with separate models for each food category (dependent variable). For each model, independent variables of interest included the reported frequency of advertisement exposure for that food category (e.g., exposure to advertisements for fast food, if the dependent variable was fast food consumption) and to one or more marketing techniques in the previous 30 days. Separate ordinal logistic regression models were used to examine the frequency of reported consumption of fruits or vegetables (0 times, 1 time, 2 times, or 3 or more times) in relation to frequency of exposure to fruit and vegetable advertisements and exposure to marketing techniques in the previous 30 days. All models were adjusted for country, age, sex, ethnicity and perceived income adequacy. To examine differences between countries, two-way interaction terms between country and either the applicable frequency of food advertisement exposure variable (e.g., frequency of exposure to fast food advertisements, if the outcome was fast food consumption) or the exposure to one or more marketing techniques variable were included in these models. Models were stratified by country when one or more significant interactions were observed, which occurred for the fruit model, vegetables model and snacks model. Analyses were conducted using IBM SPSS Statistics (Version 29.0.1.0) using the Complex Samples module and results were considered statistically significant when *p* < 0.05.

## Results

### Sociodemographic characteristics

The weighted sample characteristics of youth participants are presented in Table 1. Overall, 51.6% of participants identified as male, 65.1% were between the ages of 13 and 17 years, 69.7% identified as the ethnic majority group in their country, and 42.7% were from households where the parent/guardian reported low perceived income adequacy. Differences in sociodemographic characteristics between countries were also observed. Notably, the proportion of participants who identified as the ethnic majority group in their country ranged from 50.2% in the US to 85.9% in Chile. Moreover, 15.7% of youth from Chile were from households where it was reportedly “very difficult” to make ends meet, compared with only 7.6% of youth from the UK. Conversely, the proportion of participants from households where it was “very easy” to make ends meet ranged from 1.3% in Chile to 13.0% in the US.

**Table 1:** Sociodemographic characteristics of youth in the 2023 IFPS youth survey analytic sample (weighted estimates, *n* = 9057)

	Total (*n* = 9057)	Australia (*n* = 1032)	Canada (*n* = 2810)	Chile (*n* = 1314)	Mexico (*n* = 1409)	United Kingdom (*n* = 1167)	United States (*n* = 1325)
	% (*n*)	% (*n*)	% (*n*)	% (*n*)	% (*n*)	% (*n*)	% (*n*)
Sex							
Male	51.6 (4674)	51.6 (532)	51.8 (1455)	51.6 (678)	51.0 (719)	51.9 (605)	51.7 (685)
Female	48.4 (4383)	48.4 (500)	48.2 (1355)	48.4 (636)	49.0 (690)	48.1 (562)	48.3 (640)
Age group							
10–12 years	34.9 (3160)	37.6 (388)	35.4 (995)	33.8 (444)	34.2 (482)	35.5 (415)	32.9 (435)
13–17 years	65.1 (5897)	62.4 (644)	64.6 (1,815)	66.2 (870)	65.8 (927)	64.5 (752)	67.1 (890)
Ethnicity							
Majority	69.7 (6313)	66.6 (687)	65.6 (1,842)	85.9 (1,129)	79.4 (1,119)	74.5 (870)	50.2 (665)
Minority	30.3 (2744)	33.4 (345)	34.4 (968)	14.1 (185)	20.6 (290)	25.5 (297)	49.8 (660)
Perceived income adequacy^a^							
Very difficult	11.4 (1028)	10.9 (113)	11.7 (328)	15.7 (206)	8.4 (118)	7.6 (88)	13.1 (174)
Difficult	31.3 (2838)	27.4 (282)	30.9 (868)	39.5 (519)	31.2 (439)	29.8 (347)	28.9 (382)
Neither easy nor difficult	33.0 (2985)	30.0 (310)	33.6 (944)	35.0 (460)	37.2 (524)	35.7 (417)	24.9 (331)
Easy	17.2 (1556)	21.6 (223)	17.2 (484)	8.5 (111)	16.1 (226)	21.0 (245)	20.1 (266)
Very easy	7.2 (650)	10.0 (103)	6.6 (186)	1.3 (18)	7.2 (102)	5.9 (69)	13.0 (172)

^a^Assessed by asking parents/guardians how easy it is for them to “make ends meet” based on their total monthly income

### Food marketing exposure

In terms of frequency of exposure to food marketing in the past 30 days, 84.5% of youth reported having been exposed to advertisements for sugary drinks, ranging from 76.5% in the UK to 96.9% in Mexico (Table 2). Among the total sample, 89.0% of the overall sample reported having been exposed to fast-food advertisements in the past 30 days, ranging from 84.6% in the UK to 95.7% in Mexico. Moreover, 80.2% of the total sample reported having been exposed to advertisements for sugary cereals in the previous 30 days, ranging from 72.9% in the UK to 95.3% in Mexico. Among the overall sample, 85.2% reported exposure to advertisements for snacks in the past 30 days, ranging from 77.8% in Australia to 97.1% in Mexico. Similarly, 83.9% of the total sample reported exposure to advertisements for desserts/treats in the previous 30 days, ranging from 77.4% in Australia to 95.5% in Mexico. There was reported exposure to advertisements for fruit or vegetables in the past 30 days for 62.9% of the total sample, ranging from 52.8% in Canada to 86.8% in Mexico.

**Table 2:** Self-reported frequency of exposure to advertising of food categories and marketing techniques for less healthy foods in the past 30 days among youth aged 10–17 years in the 2023 IFPS youth survey (weighted estimates, *n* = 9057)

	Total (*n* = 9057)	Australia (*n* = 1032)	Canada (*n* = 2810)	Chile (*n* = 1314)	Mexico (*n* = 1409)	United Kingdom (*n* = 1167)	United States (*n* = 1325)
	% (*n*)	% (*n*)	% (*n*)	% (*n*)	% (*n*)	% (*n*)	% (*n*)
Frequency of exposure in past 30 days							
Ads for sugary drinks							
Never	15.5 (1402)	19.8 (204)	21.1 (593)	5.5 (72)	3.1 (44)	23.5 (274)	16.2 (215)
Less than once a week	12.9 (1170)	16.7 (172)	15.6 (438)	8.3 (109)	7.1 (100)	18.4 (215)	10.2 (135)
Once a week	15.9 (1443)	18.3 (189)	16.7 (469)	16.8 (220)	13.3 (188)	15.8 (185)	14.5 (192)
A few times a week	34.6 (3137)	28.8 (297)	32.6 (915)	38.9 (511)	38.5 (543)	33.0 (385)	36.7 (486)
Every day	15.3 (1386)	11.7 (121)	10.4 (293)	22.8 (299)	28.5 (402)	6.6 (77)	14.7 (195)
More than once a day	5.7 (518)	4.7 (47)	3.6 (102)	7.8 (102)	9.4 (132)	2.7 (31)	7.7 (102)
Ads for fast food from a restaurant							
Never	11.0 (992)	12.7 (131)	13.9 (391)	7.1 (93)	4.3 (60)	15.4 (180)	10.3 (137)
Less than once a week	10.5 (948)	12.8 (132)	11.6 (325)	10.2 (134)	7.8 (109)	12.8 (150)	7.3 (97)
Once a week	13.6 (1227)	14.2 (147)	14.4 (405)	12.3 (162)	13.0 (183)	15.8 (184)	11.0 (146)
A few times a week	34.9 (3159)	32.9 (340)	33.1 (930)	38.2 (502)	37.0 (521)	38.0 (443)	31.9 (423)
Every day	22.6 (2047)	19.2 (198)	20.7 (581)	24.5 (322)	30.4 (428)	13.9 (162)	26.9 (356)
More than once a day	7.6 (685)	8.1 (83)	6.3 (178)	7.7 (102)	7.6 (107)	4.1 (48)	12.6 (167)
Ads for sugary cereals							
Never	19.8 (1794)	29.3 (303)	25.5 (717)	10.8 (142)	4.7 (66)	27.1 (316)	18.9 (250)
Less than once a week	17.3 (1570)	19.8 (204)	19.9 (559)	14.1 (185)	11.9 (167)	21.4 (249)	15.5 (205)
Once a week	15.9 (1438)	14.8 (153)	16.3 (457)	19.8 (260)	12.9 (182)	15.9 (186)	15.1 (200)
A few times a week	29.0 (2625)	23.8 (246)	25.8 (725)	33.4 (439)	37.7 (532)	25.4 (296)	29.2 (387)
Every day	14.0 (1265)	8.6 (89)	9.4 (264)	17.2 (226)	27.2 (384)	7.7 (90)	16.0 (211)
More than once a day	4.0 (365)	3.6 (37)	3.1 (88)	4.6 (60)	5.5 (78)	2.6 (30)	5.4 (72)
Ads for snacks							
Never	14.8 (1342)	22.2 (230)	19.5 (547)	6.8 (90)	2.9 (40)	20.8 (242)	14.6 (193)
Less than once a week	13.1 (1184)	17.0 (175)	17.7 (499)	8.7 (114)	4.8 (67)	16.1 (187)	10.7 (142)
Once a week	15.3 (1384)	14.2 (147)	17.6 (494)	15.4 (202)	9.7 (137)	17.1 (200)	15.4 (204)
A few times a week	32.9 (2976)	29.8 (308)	30.4 (854)	40.3 (530)	32.9 (464)	32.6 (381)	33.2 (440)
Every day	18.2 (1648)	12.5 (129)	11.4 (320)	22.4 (294)	39.0 (550)	10.7 (125)	17.4 (230)
More than once a day	5.8 (523)	4.2 (44)	3.4 (96)	6.4 (85)	10.7 (150)	2.7 (32)	8.7 (116)
Ads for desserts/treats							
Never	16.1 (1459)	22.6 (234)	21.0 (590)	8.4 (110)	4.5 (64)	22.1 (258)	15.4 (204)
Less than once a week	13.4 (1218)	16.4 (170)	16.8 (472)	9.3 (122)	6.4 (91)	18.6 (217)	11.1 (147)
Once a week	15.9 (1444)	17.9 (184)	17.7 (498)	15.4 (202)	11.3 (159)	16.8 (196)	15.4 (204)
A few times a week	31.3 (2833)	27.7 (286)	29.4 (825)	35.5 (467)	33.4 (470)	29.3 (342)	33.4 (443)
Every day	17.4 (1578)	11.5 (118)	11.5 (323)	23.8 (313)	34.4 (485)	9.9 (116)	16.8 (223)
More than once a day	5.8 (525)	3.9 (40)	3.6 (102)	7.6 (100)	9.9 (140)	3.3 (38)	7.9 (105)
Ads for fruit or vegetables							
Never	37.1 (3363)	38.7 (400)	47.2 (1327)	31.2 (410)	13.2 (186)	45.9 (536)	38.1 (504)
Less than once a week	20.4 (1846)	20.1 (207)	20.6 (579)	22.6 (298)	19.8 (280)	20.0 (233)	18.8 (249)
Once a week	12.9 (1164)	12.6 (130)	11.7 (328)	14.3 (188)	15.6 (220)	11.2 (131)	12.6 (167)
A few times a week	18.4 (1667)	17.2 (177)	14.2 (398)	19.0 (250)	30.2 (425)	15.0 (175)	18.3 (242)
Every day	8.5 (772)	8.7 (90)	4.7 (131)	10.2 (134)	16.8 (236)	6.1 (71)	8.3 (110)
More than once a day	2.7 (245)	2.8 (29)	1.7 (47)	2.6 (35)	4.4 (62)	1.8 (21)	3.9 (52)
Exposure to marketing techniques in less healthy food or drink ads in the past 30 days							
Exposed to one or more techniques	63.6 (5758)	54.6 (563)	56.0 (1574)	81.1 (1066)	84.3 (1188)	44.0 (513)	64.5 (854)
Sports teams or athletes	35.7 (3237)	30.6 (315)	29.1 (817)	47.9 (629)	54.6 (769)	19.6 (229)	36.0 (476)
Cartoons or characters from movies or TV	34.0 (3083)	29.4 (303)	29.4 (827)	42.2 (555)	46.5 (656)	20.0 (233)	38.4 (508)
Cartoons or characters made by food companies	42.9 (3889)	36.2 (374)	38.6 (1085)	49.5 (651)	55.2 (778)	29.7 (347)	49.4 (655)
Famous people	43.3 (3919)	35.2 (364)	34.8 (978)	64.2 (844)	62.0 (873)	26.0 (303)	42.0 (556)

Additionally, as shown in Tables 2 and 63.6% of the total sample reported being exposed to less healthy food advertisements featuring one or more of the four marketing techniques examined (sports teams or athletes; cartoons or characters from movies or TV; cartoons or characters made by food companies; famous people), in the past 30 days, ranging from 44.0% in the UK to 84.3% in Mexico. Among the overall sample, featuring famous people in advertisements was the technique that youth most reported being exposed to in less healthy food advertisements (43.3%).

### Consumption of less healthy foods and fruit and vegetables

Among the total sample, 53.7% reported having consumed sugary drinks yesterday, ranging from 40.4% in the UK to 72.7% in Mexico (Table 3). Consumption of fast food the previous day was less prevalent among the overall sample at 33.3%, ranging from 21.5% in Chile to 45.0% in the US. Sugary cereals were reported to be consumed by 40.2% of the total sample the previous day, ranging from 32.5% in Australia to 56.4% in Mexico. Furthermore, 80.1% of the total sample reported having consumed snacks the day before, ranging from 76.9% in Chile to 84.4% in Mexico. Desserts/treats were reported to be consumed by 69.3% of youth in the total sample, ranging from 65.8% in Australia to 73.5% in Mexico. In terms of fruit consumption across all 6 countries, 36.0% of youth reported having consumed fruit one time during the previous day. Notably, nearly one-quarter (24.0%) of participants from Australia reported having consumed fruit three or more times in the previous day, while 22.9% of participants from the USA reported having consumed fruit zero times. Additionally, 41.1% of the total sample reported having consumed vegetables one time the previous day. Among participants from Australia, 25.0% reported having consumed vegetables three or more times the previous day, while approximately one-fifth (19.8%) of US participants reported consuming no vegetables the previous day.

**Table 3:** Youth’s self-reported consumption of less healthy foods and fruits and vegetables the day prior to the survey, presented by country (weighted estimates, *n* = 9057)

	Total (*n* = 9057)	Australia (*n* = 1032)	Canada (*n* = 2810)	Chile (*n* = 1314)	Mexico (*n* = 1409)	United Kingdom (*n* = 1167)	United States (*n* = 1325)
	*n* (%)	*n* (%)	*n* (%)	*n* (%)	*n* (%)	*n* (%)	*n* (%)
Consumption of food yesterday^a^							
Sugary drinks	53.7 (4866)	51.4 (530)	46.7 (1313)	59.1 (776)	72.7 (1025)	40.4 (472)	56.6 (750)
Fast food	33.3 (3019)	42.2 (435)	35.3 (992)	21.5 (283)	28.0 (394)	27.3 (319)	45.0 (596)
Sugary cereals	40.2 (3637)	32.5 (335)	34.2 (962)	45.9 (603)	56.4 (794)	36.8 (430)	38.7 (513)
Snacks	80.1 (7252)	77.1 (796)	78.6 (2209)	76.9 (1010)	84.4 (1189)	80.8 (943)	83.4 (1105)
Desserts/treats	69.3 (6273)	65.8 (679)	66.4 (1865)	71.5 (940)	73.5 (1035)	72.7 (848)	68.4 (906)
Frequency of fruit consumption yesterday							
0 times	17.2 (1561)	13.8 (143)	16.5 (464)	19.2 (253)	11.3 (159)	20.4 (238)	22.9 (304)
1 time	36.0 (3264)	34.4 (355)	36.6 (1,028)	34.8 (457)	39.4 (555)	35.6 (416)	34.2 (453)
2 times	28.2 (2551)	27.8 (287)	28.1 (791)	27.9 (367)	30.9 (436)	26.7 (312)	27.0 (358)
3 or more times	18.6 (1683)	24.0 (248)	18.8 (527)	18.1 (238)	18.4 (259)	17.2 (201)	15.8 (210)
Frequency of vegetable consumption yesterday							
0 times	15.6 (1414)	11.7 (121)	15.7 (440)	11.7 (154)	14.9 (210)	19.5 (227)	19.8 (262)
1 time	41.1 (3723)	40.2 (414)	37.5 (1053)	44.7 (588)	47.3 (666)	41.4 (483)	39.1 (518)
2 times	25.9 (2348)	23.1 (238)	29.4 (825)	26.5 (348)	22.9 (322)	24.3 (284)	25.0 (331)
3 or more times	17.4 (1572)	25.0 (258)	17.5 (492)	17.0 (224)	15.0 (211)	14.8 (173)	16.2 (214)

^a^Refers to the proportion and number of youth who reported having consumed each type of food the day prior to the survey

### Consumption of less healthy foods in relation to frequency of food marketing exposure and marketing technique exposure

#### Sugary drinks

In the adjusted models, youth who reported having been exposed to advertisements for sugary drinks more than once a day (AOR: 1.86; 95% CI: 1.17, 2.95), every day (AOR: 1.75; 95% CI: 1.27, 2.41), a few times a week (AOR: 1.66; 95% CI: 1.31, 2.11) or once a week (AOR: 1.60; 95% CI: 1.23, 2.09) had higher odds of reporting having consumed sugary drinks yesterday than youth who reported never having been exposed to advertisements for sugary drinks in the previous 30 days (Table 4). There were no differences in this relationship between countries (χ2 = 35.27; *p* = 0.08). Additionally, youth who reported exposure to one or more marketing techniques in advertisements for less healthy food or drinks in the previous 30 days had higher odds of reporting having consumed sugary drinks the previous day (AOR: 1.44; 95% CI: 1.21, 1.71). There were no differences between countries in terms of the relationship between marketing technique exposure and sugary drink consumption (χ2 = 8.08; *p* = 0.15).

**Table 4:** The adjusted odds of reporting consumption of less healthy food categories the day prior to the survey in relation to both self-reported frequency of exposure to advertising of these foods in the past 30 days and exposure to one or more marketing techniques among youth in Australia, Canada, Chile, Mexico, the UK and the US (weighted estimates, *n* = 9,057)

Food category consumed in previous day										
Parameter^a^	Sugary drinks		Fast food		Sugary cereals		Snacks		Desserts/treats	
	χ2, *p*-value^b^	AOR (95% CI)^b^	χ2, *p*-value^b^	AOR (95% CI)^b^	χ2, *p*-value^b^	AOR (95% CI)^b^	χ2, *p*-value^b^	AOR (95% CI)^b^	χ2, *p*-value^b^	AOR (95% CI)^b^
Frequency of exposure to advertising of that food in the past 30 days	**34.89**, ***p*** **< 0.001**		**26.18**, ***p*** **< 0.001**		**111.06**, ***p*** **< 0.001**		**53.81**, ***p*** **< 0.001**		**73.49**, ***p*** **< 0.001**	
Less than once a week vs. never		1.21 (0.93, 1.58)		1.06 (0.75, 1.50)		1.21 (0.93, 1.56)		**1.72 (1.30**,** 2.29)**		**1.56 (1.21**,** 2.03)**
Once a week vs. never		**1.60 (1.23**,** 2.09)**		**1.52 (1.10**,** 2.10)**		**1.58 (1.21**,** 2.06)**		**1.92 (1.42**,** 2.59)**		**1.74 (1.33**,** 2.27)**
A few times a week vs. never		**1.66 (1.31**,** 2.11)**		1.26 (0.95, 1.69)		**1.55 (1.21**,** 1.99)**		**2.63 (1.98**,** 3.50)**		**2.23 (1.73**,** 2.87)**
Every day vs. never		**1.75 (1.27**,** 2.41)**		**1.42 (1.03**,** 1.95)**		**2.27 (1.63**,** 3.16)**		**2.81 (1.91**,** 4.14)**		**2.20 (1.58**,** 3.07)**
More than once a day vs. never		**1.86 (1.17**,** 2.95)**		**1.96 (1.29**,** 2.97)**		**3.32 (2.03**,** 5.43)**		**4.52 (2.22**,** 9.21)**		**2.09 (1.24**,** 3.51)**
Exposure to one or more marketing techniques^c^	**37.81**, ***p*** **< 0.001**		**61.49**, ***p*** **< 0.001**		**31.58**, ***p*** **< 0.001**		**14.74**, ***p*** **< 0.001**		**15.26**, ***p*** **< 0.001**	
Exposed vs. not exposed		**1.44 (1.21**,** 1.72)**		**1.69 (1.40**,** 2.03)**		**1.26 (1.05**,** 1.51)**		0.94 (0.76, 1.16)		**1.42 (1.18**,** 1.71)**
Country x frequency of exposure to advertising	35.27, *p* = 0.08		24.56, *p* = 0.49		26.15, *p* = 0.40		**39.88**, ***p*** **= 0.03**		30.54, *p* = 0.21	
Country x exposure to one or more marketing techniques^c^	8.08, *p* = 0.15		3.63, *p* = 0.60		6.99, *p* = 0.22		**18.76**, ***p*** **= 0.002**		4.49, *p* = 0.48	

^a^The reference category is listed second

^b^Models included an interaction term between country and either frequency of exposure to advertising or exposure to one or more marketing techniques. All models were adjusted for age, sex, ethnicity and income adequacy. Boldface indicates statistical significance (*p* < 0.05)

^c^Variable derived based on youth’s self-reported exposure to one or more of the following marketing techniques: sports teams or athletes; cartoons or characters from movies or TV; cartoons or characters made by food companies; and/or famous people

#### Fast food

Participants reporting exposure to advertisements for fast food more than once a day (AOR: 1.96; 95% CI: 1.29, 2.97), every day (AOR: 1.42; 95% CI: 1.03, 1.95), or once a week (AOR: 1.52; 95% CI: 1.10, 2.10) in the previous 30 days had higher odds of reporting consumption of fast food the previous day than those reporting no exposure to fast food advertisements (Table 4). No differences were observed between countries (χ2 = 24.56; *p* = 0.49). Reporting exposure to less healthy food advertisements featuring one or more marketing techniques in the previous 30 days was associated with increased odds of consuming fast food the previous day (AOR: 1.69; 95% CI: 1.40, 2.03), but there were no differences in this relationship between countries (χ2 = 3.63; *p* = 0.60).

#### Sugary cereals

Youth who reported having been exposed to advertisements for sugary cereals more than once a day (AOR: 3.32; 95% CI: 2.03, 5.43), every day (AOR: 2.27; 95% CI: 1.63, 3.16), a few times a week (AOR: 1.55; 95% CI: 1.21, 1.99) or once a week (AOR: 1.58; 95% CI: 1.21, 2.06) had increased odds of having consumed sugary cereals the previous day than those reporting no exposure to advertisements for sugary cereals (Table 4). There were no differences in this association between countries (χ2 = 26.15, *p* = 0.40). Reporting exposure to less healthy food advertisements featuring one or more marketing techniques in the previous 30 days was associated with a greater odds of consuming sugary cereals the previous day (AOR: 1.26; 95% CI: 1.05, 1.51); there were no differences between countries (χ2 = 6.99; *p* = 0.22).

#### Snacks

Youth who reported having been exposed to advertisements for snacks more than once a day (AOR: 4.52; 95% CI: 2.22, 9.21), every day (AOR: 2.81; 95% CI: 1.91, 4.14), a few times a week (AOR: 2.63; 95% CI: 1.98, 3.50), once a week (AOR: 1.92; 95% CI: 1.42, 2.59), or less than once a week (AOR: 1.72; 95% CI: 1.30, 2.29) were more likely to reporting having consumed snacks the previous day than those reporting no exposure to advertisements (Table 4). There were differences between countries in both the frequency of salty/savoury snack advertisements (χ2 = 39.88; *p* = 0.03) and the marketing techniques (χ2 = 18.76, *p* = 0.002) to which youth were exposed in relation to snack consumption. Significant positive associations between snack food marketing exposure and consumption of snack foods the previous day were observed in Australia (χ2 = 12.52; *p* = 0.01), Chile (χ2 = 10.89; *p* = 0.03), Mexico (χ2 = 12.08; *p* = 0.02), the UK (χ2 = 12.59; *p* = 0.01), with the strongest associations observed in Canada (χ2 = 55.81; *p* < 0.001) and the USA (χ2 = 29.20; *p* < 0.001). In addition, exposure to less healthy food/drink advertisements featuring one or more marketing techniques in the previous 30 days was associated with increased odds of salty/savoury snack consumption the previous day in Australia (AOR: 1.61; 95% CI: 1.09, 2.34), Chile (AOR: 1.63; 95% CI: 1.12, 2.36) and Mexico (AOR: 2.13; 95% CI: 1.39, 3.26), but not in Canada (AOR: 0.92; 95% CI: 0.74, 1.14), the UK (AOR: 1.23; 95% CI: 0.87, 1.73) or the USA (AOR: 0.89; 95% CI: 0.62, 1.28) after adjusting for other variables, including snack food advertisement exposure. The results of models stratified by country are presented in Supplementary Table 1 (Additional file 1).

#### Desserts/treats

Youth who reported exposure to advertisements for desserts/treats more than once a day (AOR: 2.09; 95% CI: 1.24, 3.51), every day (AOR: 2.20; 95% CI: 1.58, 3.07), a few times a week (AOR: 2.23; 95% CI: 1.73, 2.87), once a week (AOR: 1.74; 95% CI: 1.33, 2.27) or less than once a week (AOR: 1.56; 95% CI: 1.21, 2.03) had higher odds than those reporting no exposure to dessert/treat advertisements to have consumed these foods the previous day (Table 4). No differences were found between countries (χ2 = 30.54; *p* = 0.21). Participants reporting exposure to less healthy food/drink advertisements featuring one or more marketing techniques in the previous 30 days had higher odds of reporting having consumed desserts/treats yesterday (AOR: 1.42; 95% CI: 1.18, 1.71), with no differences observed between countries (χ2 = 4.49; *p* = 0.48).

#### Fruits

Significant interactions were observed between country and the frequency of exposure to advertisements for fruits and vegetables in the models for fruit and vegetable consumption. Analyses were therefore stratified by country. Reporting exposure to fruit/vegetable advertisements less than once a week was associated with more frequent fruit consumption yesterday among youth in Australia (AOR: 1.43; 95% CI: 1.02, 2.00), Chile (AOR: 1.49; 95% CI: 1.08, 2.05) and Mexico (AOR: 2.11; 95% CI: 1.43, 3.11), but not in Canada (AOR: 1.15; 95% CI: 0.96, 1.37), the UK (AOR: 1.30; 95% CI: 0.99, 1.70) or the US (AOR: 1.06; 95% CI: 0.78, 1.45), compared with those who reported no fruit/vegetable advertisement exposure (Table 5). Exposure to advertisements for fruit/vegetable once a week was associated with more frequent fruit consumption the previous day in Canada (AOR: 1.50; 95% CI: 1.19, 1.87), Chile (AOR: 2.17; 95% CI: 1.57, 3.01), Mexico (AOR: 2.55; 95% CI: 1.71, 3.80) and the US (AOR: 2.18; 95% CI: 1.51, 3.14), but not in Australia (AOR: 1.38; 95% CI: 0.96, 2.00) or the UK (AOR: 1.16; 95% CI: 0.78, 1.72), compared with youth never exposed to fruit/vegetable advertisements in the past 30 days. Youth from Australia (AOR: 2.07; 95% CI: 1.45, 2.97), Canada (AOR: 1.79; 95% CI: 1.43, 2.23), Chile (AOR: 1.93; 95% CI: 1.40, 2.66), Mexico (AOR: 2.34; 95% CI: 1.63, 3.35), the UK (AOR: 2.30; 95% CI: 1.58, 3.35) and the US (AOR: 2.85; 95% CI: 2.07, 3.94) who reported exposure to fruit/vegetable advertisements a few times a week had higher odds of reporting more frequent fruit consumption yesterday, compared with those who reported no fruit/vegetable advertisement exposure. Lastly, reporting exposure to fruit/vegetable advertisements every day or more than once a day was associated with more frequent fruit consumption yesterday among youth in Australia (AOR: 2.54; 95% CI: 1.59, 4.03), Canada (AOR: 2.41; 95% CI: 1.74, 3.34), Chile (AOR: 4.06; 95% CI: 2.76, 5.97), Mexico (AOR: 4.90; 95% CI: 3.22, 7.44), the UK (AOR: 2.63; 95% CI: 1.62, 4.28) and the US (AOR: 6.81; 95% CI: 4.55, 10.20). Additionally, youth reporting exposure to less healthy food/drink marketing featuring one or more marketing techniques had higher odds of reporting having consumed fruit more times the previous day in Australia (AOR: 1.99; 95% CI: 1.52, 2.62), Mexico (AOR: 1.49; 95% CI: 1.08, 2.06), and the UK (AOR: 1.67; 95% CI: 1.30, 2.15), but not in Canada (AOR: 1.15; 95% CI: 0.99, 1.33), Chile (AOR: 1.26; 95% CI: 0.94, 1.68) or the USA (AOR: 1.08; 95% CI: 0.84, 1.39).

**Table 5:** The adjusted odds of reporting more frequent consumption of fruit the day prior to the survey in relation to both self-reported frequency of exposure to advertising of fruits and vegetables in the past 30 days and exposure to one or more marketing techniques among youth from an ordinal logistic regression model, presented by country (weighted estimates, *n* = 9057).^a^

Parameter^b^	Australia		Canada		Chile		Mexico		United Kingdom		United States	
	χ2, *p*-value^c^	AOR (95% CI)^c^	χ2, *p*-value^c^	AOR (95% CI)^c^	χ2, *p*-value^c^	AOR (95% CI)^c^	χ2, *p*-value^c^	AOR (95% CI)^c^	χ2, *p*-value^c^	AOR (95% CI)^c^	χ2, *p*-value^c^	AOR (95% CI)^c^
Frequency of exposure to advertising of fruits and vegetables in the past 30 days	**24.08**, ***p*** **< 0.001**		**49.48**, ***p*** **< 0.001**		**56.69**, ***p*** **< 0.001**		**56.45**, ***p*** **< 0.001**		**27.52**, ***p*** **< 0.001**		**115.79**, ***p*** **< 0.001**	
Less than once a week vs. never		**1.43 (1.02**,** 2.00)**		1.15 (0.96, 1.37)		**1.49 (1.08**,** 2.05)**		**2.11 (1.43**,** 3.11)**		1.30 (0.99, 1.70)		1.06 (0.78, 1.45)
Once a week vs. never		1.38 (0.96, 2.00)		**1.50 (1.19**,** 1.87)**		**2.17 (1.57**,** 3.01)**		**2.55 (1.71**,** 3.80)**		1.16 (0.78, 1.72)		**2.18 (1.51**,** 3.14)**
A few times a week vs. never		**2.07 (1.45**,** 2.97)**		**1.79 (1.43**,** 2.23)**		**1.93 (1.40**,** 2.66)**		**2.34 (1.63**,** 3.35)**		**2.30 (1.58**,** 3.35)**		**2.85 (2.07**,** 3.94)**
Every day or more than once a day vs. never^d^		**2.54 (1.59**,** 4.03)**		**2.41 (1.74**,** 3.34)**		**4.06 (2.76**,** 5.97)**		**4.90 (3.22**,** 7.44)**		**2.63 (1.62**,** 4.28)**		**6.81 (4.55**,** 10.20)**
Exposure to one or more marketing techniques^e^	**24.42**, ***p*** **< 0.001**		3.14, *p* = 0.08		2.43, *p* = 0.12		**5.84**, ***p*** **= 0.02**		**16.10**, ***p*** **< 0.001**		0.34, *p* = 0.56	
Exposed vs. not exposed		**1.99 (1.52**,** 2.62)**		1.15 (0.99, 1.33)		1.26 (0.94, 1.68)		**1.49 (1.08**,** 2.06)**		**1.67 (1.30**,** 2.15)**		1.08 (0.84, 1.39)

^a^Due to low numbers of observations at the higher end of the scale, the fruit and vegetable consumption variables were recoded as follows: 0 times; 1 time; 2 times; 3 or more times

^b^The reference category is listed second

^c^All models were adjusted for age, sex, ethnicity and income adequacy. Boldface indicates statistical significance (*p* < 0.05)

^d^The “every day” and “more than once a day” categories were combined due to low numbers of observations

^e^Variable derived based on youth’s self-reported exposure to one or more of the following marketing techniques: sports teams or athletes; cartoons or characters from movies or TV; cartoons or characters made by food companies; and/or famous people

#### Vegetables

Australian youth (AOR: 1.89; 95% CI: 1.35, 2.66) who reported exposure to fruit/vegetable advertisements less than once a week had greater odds of reporting more frequent vegetable consumption yesterday, compared with those who reported no fruit/vegetable advertisement exposure (Table 6). No significant differences were observed among youth in Canada (AOR: 0.98; 95% CI: 0.82, 1.18), Chile (AOR: 1.35; 95% CI: 0.98, 1.86), Mexico (AOR: 1.20; 95% CI: 0.84, 1.72), the UK (AOR: 1.17; 95% CI: 0.88, 1.55) or the US (AOR: 1.24; 95% CI: 0.90, 1.70). In fact, there was no association between exposure to advertisements for fruits and vegetables in the past 30 days and consumption of vegetables the previous day among youth from the UK (χ2 = 8.21; *p* = 0.08). Exposure to fruit/vegetable advertisements once a week was associated with higher odds of more frequent vegetable consumption yesterday among youth in Australia (AOR: 1.87; 95% CI: 1.30, 2.69), Canada (AOR: 1.47; 95% CI: 1.17, 1.85), Chile (AOR: 1.64; 95% CI: 1.18, 2.28), Mexico (AOR: 1.61; 95% CI: 1.04, 2.49) and the US (AOR: 2.20; 95% CI: 1.54, 3.16), compared with those who reported no exposure. Youth from Australia (AOR: 2.06; 95% CI: 1.43, 2.96), Canada (AOR: 1.53; 95% CI: 1.23, 1.90), Chile (AOR: 1.73; 95% CI: 1.23, 2.42), Mexico (AOR: 1.43; 95% CI: 1.01, 2.03) and the US (AOR: 2.41; 95% CI: 1.76, 3.29) who reported exposure to fruit/vegetable advertisements a few times a week had higher odds of reporting more frequent consumption of vegetables yesterday, compared with youth who reported no exposure to fruit/vegetable advertisements. Exposure to advertisements for fruits and vegetables every day or more than once a day was associated with greater odds of vegetable consumption the previous day among youth in Australia (AOR: 3.52; 95% CI: 2.25, 5.51), Canada (AOR: 2.30; 95% CI: 1.67, 3.17), Chile (AOR: 3.27; 95% CI: 2.14, 4.99), Mexico (AOR: 2.68; 95% CI: 1.81, 3.97) and the US (AOR: 5.01; 95% CI: 3.30, 7.60).

**Table 6:** The adjusted odds of reporting more frequent consumption of vegetables the day prior to the survey in relation to both self-reported frequency of exposure to advertising of fruits and vegetables in the past 30 days and exposure to one or more marketing techniques among youth from an ordinal regression model, presented by country (weighted estimates, *n* = 9057).^a^

Parameter^b^	Australia		Canada		Chile		Mexico		United Kingdom		United States	
	χ2, *p*-value^c^	AOR (95% CI)^c^	χ2, *p*-value^c^	AOR (95% CI)^c^	χ2, *p*-value^c^	AOR (95% CI)^c^	χ2, *p*-value^c^	AOR (95% CI)^c^	χ2, *p*-value^c^	AOR (95% CI)^c^	χ2, *p*-value^c^	AOR (95% CI)^c^
Frequency of exposure to advertising of fruits and vegetables in the past 30 days	**38.68**, ***p*** **< 0.001**		**44.64**, ***p*** **< 0.001**		**33.48**, ***p*** **< 0.001**		**28.79**, ***p*** **< 0.001**		8.21, *p* = 0.08		**75.57**, ***p*** **< 0.001**	
Less than once a week vs. never		**1.89 (1.35**,** 2.66)**		0.98 (0.82, 1.18)		1.35 (0.98, 1.86)		1.20 (0.84, 1.72)		1.17 (0.88, 1.55)		1.24 (0.90, 1.70)
Once a week vs. never		**1.87 (1.30**,** 2.69)**		**1.47 (1.17**,** 1.85)**		**1.64 (1.18**,** 2.28)**		**1.61 (1.04**,** 2.49)**		1.17 (0.80, 1.71)		**2.20 (1.54**,** 3.16)**
A few times a week vs. never		**2.06 (1.43**,** 2.96)**		**1.53 (1.23**,** 1.90)**		**1.73 (1.23**,** 2.42)**		**1.43 (1.01**,** 2.03)**		**1.59 (1.12**,** 2.27)**		**2.41 (1.76**,** 3.29)**
Every day or more than once a day vs. never^d^		**3.52 (2.25**,** 5.51)**		**2.30 (1.67**,** 3.17)**		**3.27 (2.14**,** 4.99)**		**2.68 (1.81**,** 3.97)**		1.53 (0.94, 2.48)		**5.01 (3.30**,** 7.60)**
Exposure to one or more marketing techniques^e^	**10.16**, ***p*** **= 0.001**		2.25, *p* = 0.13		1.97, *p* = 0.16		**7.62**, ***p*** **= 0.006**		**25.50**, ***p*** **< 0.001**		**7.81**, ***p*** **= 0.005**	
Exposed vs. not exposed		**1.55 (1.18**,** 2.03)**		1.12 (0.97, 1.30)		1.25 (0.92, 1.70)		**1.61 (1.15**,** 2.25)**		**1.82 (1.44**,** 2.30)**		**1.41 (1.11**,** 1.79)**

^a^Due to low numbers of observations at the higher end of the scale, the fruit and vegetable consumption variables were recoded as follows: 0 times; 1 time; 2 times; 3 or more times

^b^The reference category is listed second

^c^All models were adjusted for age, sex, ethnicity and income adequacy. Boldface indicates statistical significance (*p* < 0.05)

^d^The “every day” and “more than once a day” categories were combined due to low numbers of observations

^e^Variable derived based on youth’s self-reported exposure to one or more of the following marketing techniques: sports teams or athletes; cartoons or characters from movies or TV; cartoons or characters made by food companies; and/or famous people

Youth who reported exposure to one or more marketing techniques in less healthy food/drink advertisements were more likely to report having consumed vegetables a greater number of times yesterday in Australia (AOR: 1.55; 95%: 1.18, 2.03), Mexico (AOR: 1.61; 95% CI: 1.15, 2.25) and the UK (AOR: 1.82; 95% CI: 1.44, 2.30), but not in Canada (AOR: 1.12; 95% CI: 0.97, 1.30), Chile (AOR: 1.25; 95% CI: 0.92, 1.70) or the USA (AOR: 1.41; 95% CI: 1.11, 1.79).

## Discussion

This study provides a novel examination of the association between self-reported exposure to food marketing and dietary intake among youth in different countries. The results demonstrate high levels of less healthy food marketing exposure and regular consumption of less healthy foods, and found exposure to marketing of less healthy foods and fruits and vegetables was associated with an increased likelihood of consuming these foods among a diverse sample of youth from six countries.

More precisely, this study found frequent exposure of youth to marketing of less healthy foods in Canada, Australia, Chile, Mexico, the UK and the US, and nearly two-thirds of the total sample (63.6%) reported exposure to less healthy food advertisements featuring marketing techniques in the previous 30 days. These findings of frequent exposure to less healthy food marketing and marketing techniques are consistent with previous research (including both self-reported survey data and observational studies) that measured youth’s exposure to food marketing on television and in digital media [14, 17, 50, 51]. Interestingly, frequent exposure to less healthy food categories and to less healthy food advertisements featuring one or more marketing techniques were consistently highest in Mexico and Chile, despite these countries having mandatory food marketing restrictions [46]. However, this study was a cross-sectional analysis and did not measure less healthy food exposure pre-policy implementation in these countries and so exposure levels may have already been higher in Mexico and Chile than the other countries sampled before the regulations were in place or the population might be particularly aware of advertising due to long-term national lobbying to pass healthy food policies. There is also the fact that marketing migrates: when one medium is regulated, marketing tends to shift to unregulated media or marketing techniques [37]. Therefore, food marketing in Mexico and Chile may have shifted to media/settings not included in their national regulations (e.g., from television to digital media in Mexico). Unsurprisingly, exposures to less healthy food marketing and marketing techniques were also high for youth from countries where only voluntary industry food marketing policies exist (Australia, the US and Canada, where only the province of Quebec has legislation restricting commercial advertising). This finding is consistent with existing literature demonstrating the limited effectiveness of voluntary policies in reducing youth’s exposure to less healthy food marketing [36, 52].

Consumption of less healthy foods the previous day was high for the overall sample, particularly for snacks (80.1%) and desserts/treats (69.3%). Additionally, nearly one-fifth of the total sample reported having consumed no fruits or vegetables the previous day. There is evidence of poor diet quality among youth in the sampled countries. For example, approximately half of the total daily energy intake of Canadian children and adolescents is from ultra-processed foods, and their diets are, on average, high in nutrients recommended to limit [5, 53]. Similar evidence of poor diet quality exists for youth from Australia [54], Chile [55], Mexico [56], the UK [57] and the US [58]. Differences in food consumption observed between countries are likely related to their national food environments, as well as the dietary patterns and norms of specific countries. For example, fast food consumption in the previous day was most common among American youth, which is expected given the US has historically been the global leader in fast food consumption and that fast-food restaurants are ubiquitous in most American neighbourhoods [59, 60]. Similarly, sugary drink consumption in the previous day was lowest among youth from the UK, which may be related to the country’s sugary drink levy implemented in 2018 [61].

These results of this study align with a consistent and growing body of evidence of the association between food marketing exposure and consumption of marketed foods among youth [10, 25, 27, 29, 31]. To our knowledge, this was among the first studies to provide a comparison of this relationship between countries. Previous research by the IFPS team demonstrated that exposure to marketing of fast food and sugary drinks was consistently and positively associated with fast food and sugary drink intakes, respectively, among youth from the same six countries as the present study (Canada, Australia, Chile, Mexico, the UK and the US), which was also documented in our study [30, 32]. In fact, we found positive associations between youth’s fast food, sugary drink, sugary cereal or dessert/treat consumption in the previous day and their frequency of exposure to advertisements for these foods, and with exposure to less healthy food advertisements featuring one or more marketing techniques, in the previous 30 days for all six countries. There were, however, some differences in these relationships between countries for snacks, fruits and vegetables. For example, despite a positive association between frequency of exposure to fruit/vegetable advertisements and vegetable consumption in all 5 other countries, no significant relationship was observed for youth from the UK for vegetable consumption. Moreover, there were associations between exposure to less healthy food advertisements featuring marketing techniques and fruit and vegetable consumption in some countries (Australia, Mexico, UK), but not others (Canada, Chile), with the US showing an association for vegetables but not for fruit. Most research on the impacts of food marketing has focused on the impacts of less healthy food marketing on consumption (and related outcomes such as food perceptions, preferences and purchases) [36]. The impacts of fruit and vegetable marketing have been less well studied [33–35]. Consistent with other studies, our findings suggest that more frequent exposure to fruit and vegetable marketing may be more strongly associated consumption of these foods. Therefore, while marketing of less healthy foods is known to be detrimental to youth’s health, marketing fruits and vegetables may be an effective strategy to promote fruit and vegetable consumption, a food group that is lacking in the diets of many children and adolescents [62]. This is likely to be even more effective if less healthy foods and beverages are not marketed simultaneously. Fruits and vegetables are marketed to youth far less frequently than more processed food and beverage products, likely at least in part because of perceived lower profitability and smaller marketing budgets for these products, and limited options for marketing techniques (e.g., due to a lack of product packaging) [63].

Findings of this study underscore the need for countries to implement mandatory restrictions on food marketing to youth – and potentially, to strengthen existing restrictions – to help limit youth’s exposure to less healthy food marketing. Of the six countries included in this study, four (Australia, Canada, UK, USA) are currently limited to voluntary industry policies concerning food marketing to children; the UK is, however, expected to implement food marketing restrictions in TV and digital media starting in January 2026 [46]. Of the sampled countries, only Chile, Mexico and the UK currently have legislation to limit less healthy food marketing [46]. However, exposure was high for youth from these countries, which may be related to loopholes in these national policies, including that they do not extend to children above a certain age, are limited to audiences comprised of a certain percentage of children, and only apply to certain media/settings [46]. The current UK regulations are particularly limited in that they only apply to television programming aimed at children aged 4–15 years (where 20% or more of the audience consists of this age group), and have resulted in unchanged exposure of children to unhealthy food advertisements and increased exposure of all viewers to these ads [18, 44]. Best practices from the WHO recommend food marketing policies that are mandatory, applicable to children of all ages (i.e., up to age 18), comprehensive across media/settings where youth are exposed to food marketing, include restrictions on the use of persuasive marketing techniques, and based on a government-led nutrient profile model to determine the healthfulness of foods for marketing to youth to successfully reduce the power and impact of food marketing [36]. Moreover, particularly with the continuing growth of digital media that transcends national borders, it is important for countries around the world to be consistent in their policy development, implementation and enforcement [36]. There is also a need for research to further examine the impact of food marketing exposure on consumption, as well as food and brand perception, preferences and purchases or purchase requests. Greater evaluation of marketing exposures and impacts is important as a growing number of countries are proposing and implementing policies to restrict food marketing to children [36]. Evidence from other countries may help prompt and inform the development of national food marketing restrictions.

### Strengths and limitations

This study is strengthened by its large and diverse sample from six countries and the use of consistent survey measures between countries. These strengths, combined with our use of post-stratification weights, increased the representativeness of our sample and generalizability of our findings. Moreover, the fact that our measures of food marketing exposure were not limited to specific media or settings enabled the reporting of associations based on a variety of exposures. Our work is, however, not without limitations. Firstly, our results are based on self-reported data for all measures, including exposure to food advertisements and marketing techniques and consumption of different types of foods. These variables may be subject to recall bias and misreporting. Youth, particularly younger children, may not have recognized all forms of marketing and have inadvertently under-estimated their exposure. However, these errors are non-differential, and the estimated odds ratio will likely be biased toward the null. Notably, the survey measure asked about “advertisements” specifically; this may have limited the forms of marketing considered by respondents in answering the question. Self-reported exposure has, however, been shown to be highly correlated with objective exposure data [64–66]. In comparison with objective measures of marketing exposure (e.g., studies using screen capture methodology), self-reported data are far less costly and labour-intensive to collect, which facilitates monitoring of youth’s exposure to unhealthy food marketing more regularly [38].

An additional limitation is that the exposure variables did not measure how appealing youth found the food marketing to which they were exposed. The advertising exposure and marketing techniques variables may also measure overlapping components of exposure. In a sensitivity analysis, models were run with each marketing variable removed to examine changes in the effect of the remaining marketing variable. In most models, the effect was very similar to the models with both variables, except for certain individual country models with salty/savoury snacks, fruit or vegetable consumption as the outcome variable, where marketing techniques became associated with the outcome when it was the only marketing variable in the model. Although there may be no effect of techniques after accounting for exposure, there is an effect of techniques in the absence of the exposure variable. Due to some overlap in the effects, the inclusion of exposure in the model attenuates the association between techniques and the outcome. Furthermore, participants may have misinterpreted some food categories, and the exact foods considered to fall into these categories may vary between countries. These results also do not reflect participants’ typical consumption patterns, and the food consumption variables did not capture information about the dietary patterns, diet quality, quantities of foods consumed or the number of times the food was consumed in the day preceding the survey (with the exception of the fruit and vegetable consumption variables, which included response options ranging from 0 times/day to 10 + times/day). Other limitations include the narrow range of marketing techniques examined, the potential for residual confounding and the fact that it was not possible to establish causality or direction of associations due to the cross-sectional design of this study. Our study is also somewhat limited by using a consumer panel, which are typically dominated by participants of higher SES identifying as the ethnic majority group. Moreover, the Canadian sample included participants from two policy environments: one with government regulations concerning food marketing to children (Quebec), and one limited to voluntary industry self-regulatory commitments in this area (all other provinces). Lastly, respondents were recruited using nonprobability-based sampling; therefore, although the data were weighted by age group, sex, region, and ethnicity (except in Canada), the findings do not provide nationally representative estimates.

## Conclusions

This study found consistent positive associations between frequency of exposure to advertisements for less healthy foods and consumption of these foods in a sample of youth from six countries. Exposure to less healthy food advertisements featuring marketing techniques was also associated with increased odds of having consumed less healthy foods across countries. Evidence of the exposure-consumption relationship was also largely consistent across countries for fruits and vegetables. The high levels of less healthy food marketing exposure and less healthy food consumption observed in this study - and the consistency of these findings across countries – highlights the need for all countries to implement mandatory, comprehensive and evidence-informed restrictions on food marketing to youth.

## Supplementary Information

Supplementary Material 1.

## Data Availability

The datasets used and/or analyzed during the current study are available from the corresponding author on reasonable request.

## Abbreviations

IFPS: International Food Policy Study
UK: United Kingdom
US: United States
WHO: World Health Organization
