# Supplementary material for: The association between exposure to food marketing and dietary intake among youth in six countries
Source: Int J Behav Nutr Phys Act. 2025 Oct 21;22:130. doi: 10.1186/s12966-025-01828-2 (PMC12539036; doi:10.1186/s12966-025-01828-2)
Supplement: Supplementary file 1 — Supplementary Material 1. [file 12966_2025_1828_MOESM1_ESM.pdf]

ADDITIONAL FILE 1

TITLE: The association between exposure to food marketing and dietary intake among youth in six countries

AUTHORS: Laura Vergeer[1], Grace Gillis[1], Vicki L. Rynard[2], Lana Vanderlee[3], Christine M. White[2], Claudia Nieto[4], David Hammond[2], Monique Potvin Kent[1]

AFFILIATIONS:

[1]School of Epidemiology and Public Health, Faculty of Medicine, University of Ottawa, Ottawa, ON, Canada

[2]School of Public Health Sciences, University of Waterloo, Waterloo, ON, Canada,

[3]École de Nutrition, Centre de Nutrition, santé et société (NUTRISS), Université Laval, Québec City, Québec, Canada

[4]Department of Public Health, Faculty of Medicine, National Autonomous University of Mexico, Mexico City, Mexico

CORRESPONDING AUTHOR: Monique Potvin Kent (monique.potvinkent@uottawa.ca)

Supplementary Table 1. The odds of reporting more frequent consumption of snacks the day prior to the survey in relation to both self-reported frequency of exposure to advertising of snacks in the past 30 days and exposure to one or more marketing techniques among youth, presented by country (weighted estimates, n=9,057).[a]

Parameter[a]	Australia		Canada		Chile		Mexico		United Kingdom		United States	
	χ2, p-value[b]	AOR (95% CI)[b]	χ2, p-value[b]	AOR (95% CI)[b]	χ2, p-value[b]	AOR (95% CI)[b]	χ2, p-value[b]	AOR (95% CI)[b]	χ2, p-value[b]	AOR (95% CI)[b]	χ2, p-value[b]	AOR (95% CI)[b]
Frequency of exposure to advertising of snacks in the past 30 days	**12.52, p=0.01**		**55.81 p<0.001**		**10.89, p=0.03**		**12.08, p=0.02**		**12.59, p=0.01**		**29.20, p<0.001**	
Less than once a week vs never		**2.13 (1.26, 3.61)**		**1.72 (1.29, 2.30)**		1.81 (0.91, 3.60)		0.72 (0.27, 1.96)		1.51 (0.93, 2.46)		1.55 (0.88, 2.73)
Once a week vs. never		**1.92 (1.11, 3.31)**		**1.94 (1.44, 2.63)**		**2.44 (1.27, 4.67)**		1.07 (0.42, 2.73)		1.50 (0.93, 2.41)		**2.46 (1.39, 4.33)**
A few times a week vs. never		**1.98 (1.23, 3.21)**		**2.65 (1.99, 3.53)**		1.43 (0.83, 2.49)		1.24 (0.53, 2.93)		**1.87 (1.22, 2.87)**		**2.89 (1.78, 4.68)**
Every day or more than once a day vs. never[c]		1.39 (0.81, 2.41)		**3.13 (2.17, 4.51)**		**2.05 (1.13, 3.71)**		2.03 (0.85, 4.82)		**2.58 (1.40, 4.75)**		**3.89 (2.22, 6.82)**
Exposure to one or more marketing techniques[d]	**5.85, p=0.02**		0.55, p=0.46		**6.63, p=0.01**		**12.30, p<0.001**		1.42, p=0.23		0.41, p=0.53	
Exposed vs. not exposed		**1.61 (1.09, 2.34)**		0.92 (0.74, 1.14)		**1.63 (1.12, 2.36)**		**2.13 (1.39, 3.26)**		1.23 (0.87, 1.73)		0.89 (0.62, 1.28)

[a]The reference category is listed second. [b]All models were adjusted for age, sex, ethnicity and income adequacy. Boldface indicates statistical significance (p<0.05). [c]The "every day" and "more than once a day" categories were combined due to low numbers of observations. [d]Variable derived based on youth's self-reported exposure to one or more of the following marketing techniques: sports teams or athletes; cartoons or characters from movies or TV; cartoons or characters made by food companies; and/or famous people.
